# Dietary supplementation with spray-dried animal plasma improves vaccine protection in aged mice

**DOI:** 10.3389/fnut.2023.1050961

**Published:** 2023-03-24

**Authors:** Lluïsa Miró, Cristina Rosell-Cardona, Concepció Amat, Javier Polo, Miquel Moretó, Anna Pérez-Bosque

**Affiliations:** ^1^Departament de Bioquímica i Fisiologia, Facultat de Farmàcia i Ciències de l’Alimentació and Institut de Recerca en Nutrició i Seguretat Alimentària (INSA-UB), Universitat de Barcelona (UB), Barcelona, Spain; ^2^APC-Europe S.L.U., Granollers, Spain

**Keywords:** senescence, immune protection, immunization, dietary supplementation, spray-dried porcine plasma

## Abstract

**Background:**

Senescence is characterized by an aggravated inflammatory state that reduces vaccine responsiveness. Dietary supplementation with spray-dried porcine plasma (SDP) exerts anti-inflammatory effects in different mucosal areas. We aimed to determine if the anti-inflammatory properties of SDP improve the efficiency of immunization in senescent animals.

**Methods:**

Experiments were performed in 2-month-old and 6-month-old male SAMP8 mice fed control or SDP (8%) feeds for 4  months. The mice received nasal doses of 2.5  μg of *Staphylococcus aureus* enterotoxin B (SEB) or vehicle every 15  days (i.e., 3 times). Fifteen days after the last dose, a lethal shock was induced by intraperitoneal administration of SEB and LPS.

**Results:**

Immunization increased anti-SEB IgA in intestinal and bronchoalveolar fluid (*p* < 0.05). After the lethal shock, all immunized aged mice that were supplemented with SDP survived, in contrast to only 66% of those fed the control feed (*p* < 0.05). Moreover, after the lethal challenge, aged mice showed higher expression levels of pro-inflammatory cytokines (*Il-6*, *Tnf-α*, *Ifn-γ*, and *Il-1β*) in jejunal and (*Tnf-α*, and *Il-1β*) in lung tissues (*p* < 0.05), which were reduced by SDP supplementation (*p* < 0.05). Furthermore, in senescent mice, SDP supplementation augmented *Il-4* and *Il-10* expression in both tissues (*p* < 0.05).

**Conclusion:**

SDP reduces the mucosal inflammation associated with aging, improving vaccine protection in senescent mice.

## 1. Introduction

During senescence, there is increased activation of the immune system that impairs pathogen-specific responses. These age-associated immune changes are known as immunosenescence and promote a pro-inflammatory profile called inflammaging (1). Moreover, this condition is characterized by a decline in immune competence and reduced capacity to generate tolerance to innocuous antigens (2). This means that aging compromises the immune protection of the gut mucosa. Elderly people experience a progressive deterioration that affects their ability to generate both innate and adaptive responses to infections, as well as exhibiting reduced vaccination efficiency (3). Therefore, they are more predisposed to the development of complications from infections, which lead to prolonged hospitalization and an increased risk of morbidity and mortality (4). Furthermore, Sasaki et al. (5) found that aged people produce comparatively fewer antibodies than young people after immunization and that these antibodies are of a lower quality in terms of neutralizing capacity. Likewise, other researchers have shown that the response to vaccination in the elderly involves the production of more non-specific antibodies than in young people (6).

Dietary supplementation with probiotics, prebiotics, or protein concentrates are widely used to modulate the immune response and cytokine expression of the gut-associated lymphoid tissue (GALT) (7, 8). Dietary supplementation with spray-dried porcine plasma (SDP) attenuates GALT activation and reduces mucosal inflammation and thereby preserves the intestinal barrier integrity in weanling rodents challenged with *S. aureus* enterotoxin B (SEB) (9, 10). Furthermore, in senescent SAMP8 mice, SDP supplementation ameliorates the persistent low-intensity intestinal inflammation associated with aging, leading to an enhanced immune response to SEB (11). The effects of SDP on intestinal inflammation are also reflected in reduced peripheral inflammation in senescent mice, which is associated with lower neuroinflammation (12, 13) and a reduced presence of Alzheimer’s disease markers (14).

Therefore, the aim of the present work was to assess whether the anti-inflammatory properties of SDP can improve vaccine protection in senescent mice. SAMP8 are mice prone to accelerated senescence and are commonly used in aging studies (15). Moreover, SAMP8 mice show alterations in the immune system, such as impaired B cell function and a reduced IgG1 concentration in serum, which may dampen the response to vaccination (16). Therefore, SAMP8 mice are an excellent model for studying the effects of SDP supplementation in immunized senescent mice.

## 2. Materials and methods

### 2.1. Animals and feeds

Male mice of the SAMP8 strain (prone to accelerated senescence) were obtained from Envigo (Bresso, Italy). The colony generated was maintained at the animal facility of the Faculty of Pharmacy and Food Science of the University of Barcelona. Animals were housed under constant temperature and humidity conditions, with a 12-h:12-h light:dark cycle. All animal experiments were performed following the Guide for the Care and Use of Laboratory Animals, and the protocols used in this study were approved by the Ethics Committee for Animal Experimentation of the Universitat de Barcelona and the Catalan government (ref. 484/16 and 9272, respectively). Animals were housed individually and fed experimental feeds (control or SDP) for 4 months, from 2 months of age to 6 months.

The second of the two experimental feeds (SDP) comprised feed supplemented with 8% SDP. SDP is a feed ingredient obtained by the centrifugation of blood from healthy pigs, which is then spray-dried to obtain a stable powder. SDP maintains the native structure of its proteins, as confirmed by immunoelectrophoresis and western blotting (17). Both control and SDP feeds were balanced for energy, carbon and total nitrogen. Both experimental feeds showed a similar essential amino acid composition, and lysine and methionine were formulated to meet National Research Council requirements (18) for laboratory animals (Table 1). The nitrogen content of the control feed was adjusted with milk protein. All feeds were prepared by Envigo.

**Table 1:** Composition of experimental feeds.

Ingredient (g/kg)	Control feed	SDP^1^ feed
	g/kg	
SDP^1^	–	80
Dried skim milk	530.7	340.4
Corn starch	199.3	308.7
Sucrose	94.5	94.5
Soybean oil	70	70
Cellulose	50	50
AIN-93-G-MX (94046)^2^	35	35
AIN-93 VX (94047)^2^	15	15
DL-Methionine	2.5	3.2
Choline bitartrate	3	3

1 SDP (spray-dried porcine plasma) is provided by APC-Europe, Spain.

2 AIN-93 VX, vitamin mix; AIN-93 MX, mineral mix, both were provided by Envigo (Bresso, Italy).

### 2.2. Experimental design

The study was divided into two experiments. The first focused on determining how aging and SDP supplementation affect variables involved in immunization while the second tested whether SDP supplementation modified the efficacy of immunization after a lethal shock.

**Figure 1:**
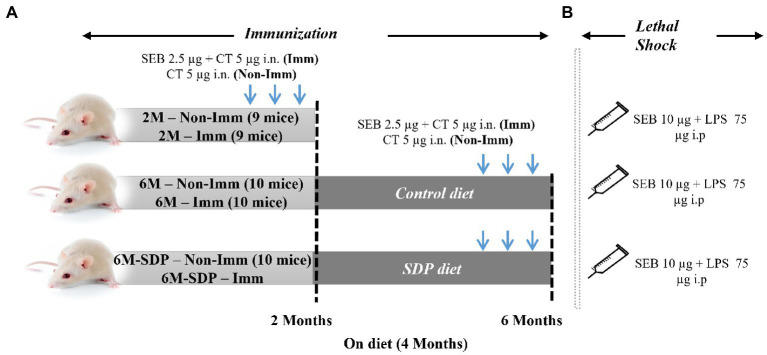
Experimental design. Mice were distributed into various experimental groups: non-immunized young mice fed standard feed (2 M), immunized young mice fed standard feed (2 M-Imm), non-immunized senescent mice fed control feed (6 M), immunized senescent mice fed control feed (6 M-Imm), non-immunized senescent mice fed SDP feed (6 M-SDP), and immunized senescent mice fed SDP feed (6 M-SDP-Imm). In Experiment 1 **(A)**, the immunized (**Imm**) mice received three doses of 2.5  μg of *Staphylococcus aureus* enterotoxin B (SEB) and 5  μg of cholera toxin (CT) at 15-day intervals. The young group received the immunizations on day 21 (at weaning), at 1 month old, and at 1.5  months old and were euthanized at 2  months of age. The senescent groups received the immunizations at 4.5, 5, and 5.5  months old and were euthanized at 6  months old. The non-immunized (**Non-Imm**) mice received only 5  μg of CT as a vehicle. Senescent mice were fed the experimental diets for 4  months, between 2 and 6  months old. Experiment 2 **(B)** was performed to test the protective capacity of the immunization. In this experimental set, a lethal shock was induced 15  days after the last immunization *via* the intraperitoneal administration of 10  μg SEB and 75  μg LPS. There were 9–10 animals in each grouped.

In both experiments, the animals were separated into three groups: 2M, 2-month-old mice (young reference group); 6M, 6-month-old senescent mice that had consumed the control feed; and 6M-SDP, 6-month-old senescent mice that had consumed the SDP feed for the previous 4 months (Figure 1). The feed intake and changes in body weight of the mice were monitored throughout the experimental period.

#### 2.2.1. Experiment 1: Immunization procedure

The three experimental groups (2M, 6M, and 6M-SDP) were also divided into immunized (Imm) and non-immunized (Non-Imm) animals. Therefore, the experimental groups in this experiment were: non-immunized young mice (2M), immunized young mice (2M-Imm), non-immunized senescent mice fed control feed (6M), immunized senescent mice fed control feed (6M-Imm), non-immunized senescent mice fed SDP feed (6M-SDP), and immunized senescent mice fed SDP feed (6M-SDP-Imm).

To perform the immunization procedure, mice were intranasally administered 2.5 μg of SEB (Toxin Technology, Inc., Sarasota, FL, United States) with 5 μg of cholera toxin (CT; Sigma-Aldrich, St. Louis, MI, United States) as immune adjuvant. Non-immunized mice received CT alone. All immunized animals received three vaccination doses every 2 weeks, as described previously (19). The young group was immunized on day 21 (at weaning), at 1 month old, and at 1.5 months old and were euthanized at 2 months of age. The older groups received the immunizations at 4.5, 5, and 5.5 months old and were euthanized at 6 months old.

#### 2.2.2. Experiment 2: Lethal shock procedure

To evaluate the protective efficiency of immunization in senescent mice and the possible effect of SDP supplementation, mice were challenged with a lethal shock comprising SEB and lipopolysaccharide (LPS) as described previously (20). Briefly, 15 days after the last immunization, when the animals were 2 and 6 months old, they were intraperitoneally administered 10 μg SEB and 75 μg LPS (Sigma-Aldrich). Both compounds were administered separately: the SEB enterotoxin was administered first, followed 3 h later by the LPS.

### 2.3. Sample collection and processing

At the end of the experimental period, mice were anesthetized with xylazine/ketamine. Blood was directly collected from the heart and the mice were euthanized during the intervention. Once obtained, samples of serum and jejunal and lung tissue were quickly frozen at −80°C for further analysis.

The intestinal fluid was obtained after the small intestinal lumen was washed with phosphate-buffered saline (PBS). The liquid obtained was clarified by centrifugation and stored at −80°C for further analysis.

### 2.4. Peyer’s patch cell isolation

Peyer’s patches were obtained as previously described (11). Immune cell recruitment in PP was assessed by counting total cells obtained. Cell viability exceeded 90% in all cases.

### 2.5. Bronchoalveolar lavage fluid

Bronchoalveolar lavage fluid (BALF) was obtained as previously described (21). BALF suspensions were centrifuged at 950*g* for 10 min to pellet BALF cells. Supernatants were stored at −80°C for the further measurement of immunoglobulins (Igs) while the pelleted cells were suspended in PBS with 5% fetal calf serum (GIBCO, Grand Island, NY, United States). Immune cell recruitment in BALF was assessed by counting total cells obtained. In all cases, cell viability exceeded 95%.

### 2.6. Cell staining

For cell staining, we used 1.5 × 10^5^ cells, as described previously by Miró et al. (11). The following conjugated primary antibodies were used: anti-CD19 to stain B lymphocytes (clone eBio1D3; eBioscience, San Diego, CA, United States) and biotin-conjugated anti-IgA (clone RMA-1; BioLegend, San Diego, CA, United States). The stained cells were quantified in a Gallios Flow Cytometer (Beckman Coulter, Miami, FL, United States), located in the Cytometry Unit of the Scientific-Technical Services of the Barcelona Science Park. The results were analyzed using Flowjo® software (version 7.6.5; Treestar Inc., Ashland, OR, United States). Lymphocytes and non-lymphocytic leukocytes were separated by forward/side scatter.

### 2.7. Immunoglobulin concentration

Total secreted IgA and anti-SEB-specific IgA were determined in intestinal fluid and BALF by sandwich enzyme-linked immunosorbent assay (ELISA). Briefly, 96-well polystyrene plates (Nunc Maxisorp; Nunc, Wiesbaden, Germany) were coated with anti-mouse IgA monoclonal antibody (1 μg/mL; Sigma-Aldrich) in PBS for quantification of the total IgA concentration or with SEB (0.5 μg/mL; Toxin Technology, Inc.) in PBS for measurement of the anti-SEB-specific IgA titer. After the plates were washed, samples and standards were added and incubated for 2 h. Mouse IgA (Bethyl, Montgomery, TX, United States) was used as a standard for the total IgA determination and an SEB-specific IgA standard curve was derived from serial dilutions of a pool of SEB-positive samples. Horseradish peroxidase-conjugated goat anti-mouse IgA (Bethyl) was used as the detection antibody.

Total IgG and SEB-specific IgG concentrations were determined in serum. As capture antibodies, we used goat anti-mouse IgG (1 μg/mL; Sigma-Aldrich) to quantify the total IgG concentration or SEB (0.125 μg/mL; Toxin Technology, Inc.) in PBS to measure the SEB-specific IgG concentration. After the plates were washed, samples and standards were added and incubated for 2 h. As standard, mouse IgG (Sigma-Aldrich) was used for total IgG determination while SEB antibody (GeneTex, Irvine, CA, United States) was used to quantitate the SEB-specific IgG concentration. Horseradish peroxidase-conjugated goat anti-mouse IgG (Sigma-Aldrich) was used as the detection antibody.

In all cases, o-phenylenediamine (OPD, 0.4 mg/mL; Sigma-Aldrich) was used as HRP substrate, and the color intensity was measured at 492 nm in a microplate reader (Sunrise, Tecan, Männedorf, Zurich, Switzerland).

### 2.8. Real-time polymerase chain reaction

RNA extraction and reverse transcription were carried out as previously described (22). RNA quality and quantity were assessed by spectrophotometry (NanoDrop ND-1000; Thermo Fisher Scientific, Waltham, MA, United States) and its integrity was determined with an Agilent 2,100 Bioanalyzer (Agilent Technologies Inc., Waldbronn, Germany). In all cases, the RNA integrity was ≥9 and the A260/280 ratio was between 1.96 and 2.02. Total RNA was reverse-transcribed using an iScript™ cDNA Synthesis Kit (Bio-Rad, Hercules, CA, United States). For real-time PCR determinations, we used a cDNA template in a 20-μL reaction solution containing 0.2 μmol/L of each primer and SsoAdvanced™ Universal SYBR^®^ Green Supermix (Bio-Rad). The primers used are listed in Table 2. Real-time PCR was performed on a MiniOpticon Real-Time PCR System (Bio-Rad). Each PCR run included duplicates of reverse transcript cDNA for each sample and negative controls (reverse transcription-free samples, RNA-free sample). Quantification of the target gene transcripts was conducted using glucuronidase beta (*Gusβ*) gene expression as reference and was performed with the 2^−ΔΔCT^ method (23). Product fidelity was confirmed by melting curve analysis.

**Table 2:** Primers used for analysis of cytokine expression.

Primer	Forward (5′-3′)	Reverse (5′-3′)	Fragment size	Accession number
*Ifn-γ*	CCTTCTTCAGCAACAGCAAGGCG	CTTGGCGCTGGACCTGTGGG	87 bp	NM_008337.3
*Il-1β*	TGTGAAATGCCACCTTTTGA	GGTCAAAGGTTTGGAAGCAG	94 bp	NM_008361.4
*Il-4*	CGGAGATGGATGTGCCAAAC	AAGCACCTTGGAAGCCCTA	83 bp	NM_021283.2
*Il-6*	ACCAGAGGAAATTTTCAATAGGC	TGATGCACTTGCAGAAAACA	109 bp	NM_031168.1
*Il-10*	GGCGCTGTCATCGATTTCTCCCC	TGGCCTTGTAGACACCTTGGTCTT	102 bp	NM_010548.2
*Gusβ*	CCGATTATCCAGAGCGAGTATG	CTCAGCGGTGACTGGTTCG	97 bp	NM_010368.1
*Tnf-α*	CCACCACGCTCTTCTGTCTAC	AGGGTCTGGGCCATAGAACT	103 bp	NM_013693.2

Ifn-γ, interferon-gamma; Il, interleukin; Gusβ, glucuronidase beta; Tnf-α, tumor necrosis factor α.

### 2.9. Statistical analysis

Results are presented as mean ± SEM. All data were analyzed with the Levene test to assess homogeneity of variance and with the Shapiro–Wilk test to assess distribution. Homogeneous and normally distributed data were analyzed with parametric two-way analysis of variance (ANOVA) or with three-way ANOVA followed by Fisher’s least significant difference (LSD) *post hoc* test using GraphPad Prism^®^ software v.9.3.1 (GraphPad Software, Inc., La Jolla, CA, United States). The effect of immunization on leukocyte recruitment, B cell percentage, and Ig concentration were analyzed by two-way ANOVA using the factors immunization (Imm vs. Non-Imm) and age-feed group (i.e., 2 M, 6 M, and 6 M-SDP). The effects of immunization and SDP supplementation on body weight and food intake were analyzed by three-way ANOVA with repeated measures, using the factors immunization (Imm vs. Non-Imm), age-feed group (2 M, 6 M, and 6 M-SDP), and time. Survival after shock induction was analyzed with a log-rank (Mantel-Cox) test. To evaluate the effects of lethal shock on cytokine expression, we considered the factors immunization (Imm vs. Non-Imm) and age-feed (2 M, 6 M, and 6 M-SDP). Differences were considered significant at *p* < 0.05.

## 3. Results

### 3.1. Food intake and changes in body weight

**Figure 2:**
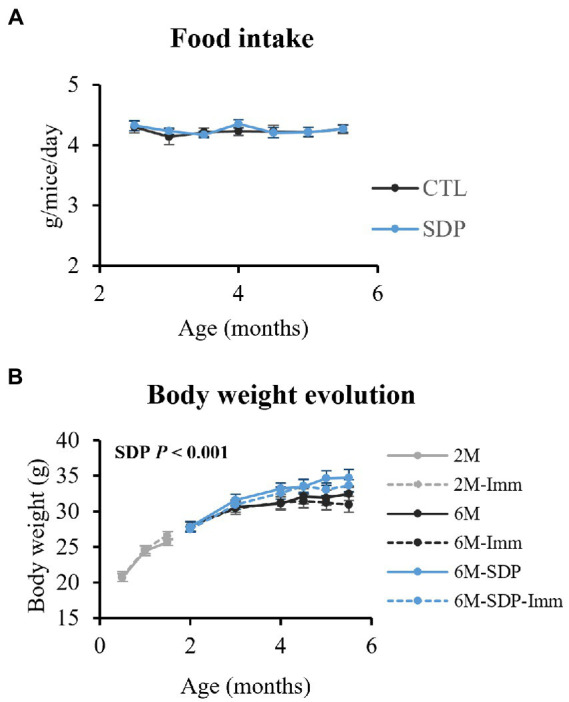
Food intake **(A)** and changes over time in body weight **(B)**. Results are expressed as means ± SEMs (*n* = 18–20 animals for food intake and *n* = 9–10 animals for changes in body weight). Statistics: for food intake, two-way ANOVA (feed and time; Fisher multiple comparison test); changes over time in body weight, three-way ANOVA (feed, immunization, and time; Fisher multiple comparison test). Imm, immunized mice; Non-Imm, non-immunized mice; SDP, spray-dried porcine plasma.

Immunization with SEB did not modify food intake or changes over time in body weight (Figure 2). On the other hand, SDP supplementation increased body weight (*p* < 0.001), without changing food intake.

### 3.2. Experiment 1: SEB immunization

#### 3.2.1. Effects of immunization on B lymphocytes from Peyer’s patches

**Figure 3:**
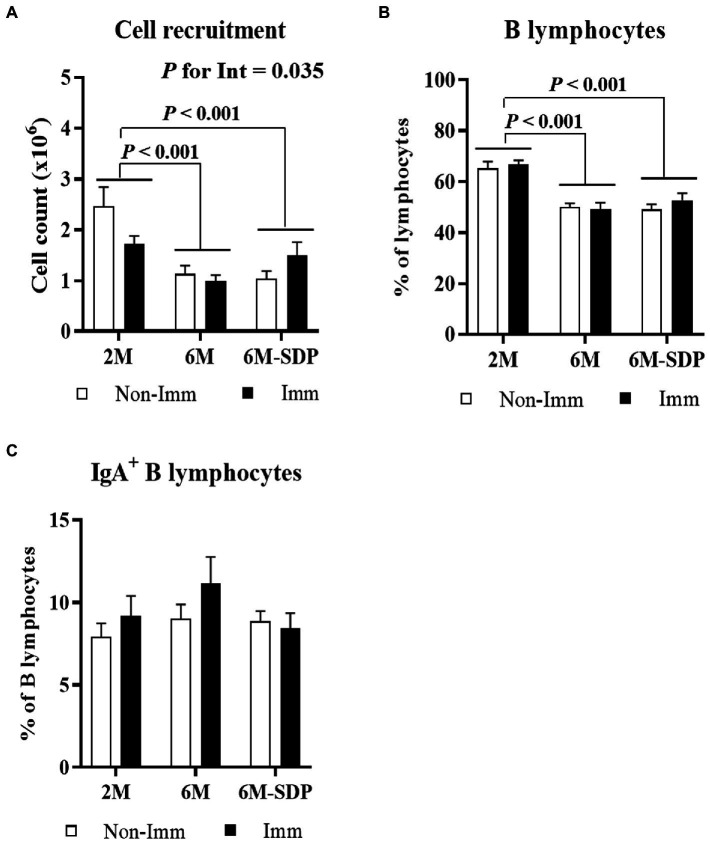
Cell recruitment **(A)**, percentage of B lymphocytes **(B)**, and percentage of IgA^+^ B lymphocytes **(C)** in Peyer’s patches. Open bars represent non-immunized mice (**Non-Imm**); solid bars represent immunized mice (**Imm**). Results are expressed as means ± SEMs (*n* = 9–10 animals). Statistics: two-way ANOVA (age-feed and immunization factors; Fisher multiple comparison test). Int indicates the interaction between immunization and age-feed factors. IgA, immunoglobulin A; SDP, spray-dried porcine plasma.

Senescent mice showed a reduced leukocyte count in Peyer’s patches compared with young animals (6M vs. 2M; *p* < 0.001, Figure 3A). Although there was no global effect of SEB immunization, there was an interaction between immunization and mouse age (*p* = 0.035) because immunization reduced cell recruitment in young mice and had no effect on aged mice. Senescence also reduced the percentage of B lymphocytes in Peyer’s patches (6M and 6M-SDP vs. 2M; *p* < 0.005, Figure 3B). Neither SDP supplementation nor immunization modified this percentage. The percentage of IgA^+^ B lymphocytes was not modified by age, SDP supplementation, or immunization (Figure 3C).

#### 3.2.2. Effects of immunization on B lymphocytes from BALF

**Figure 4:**
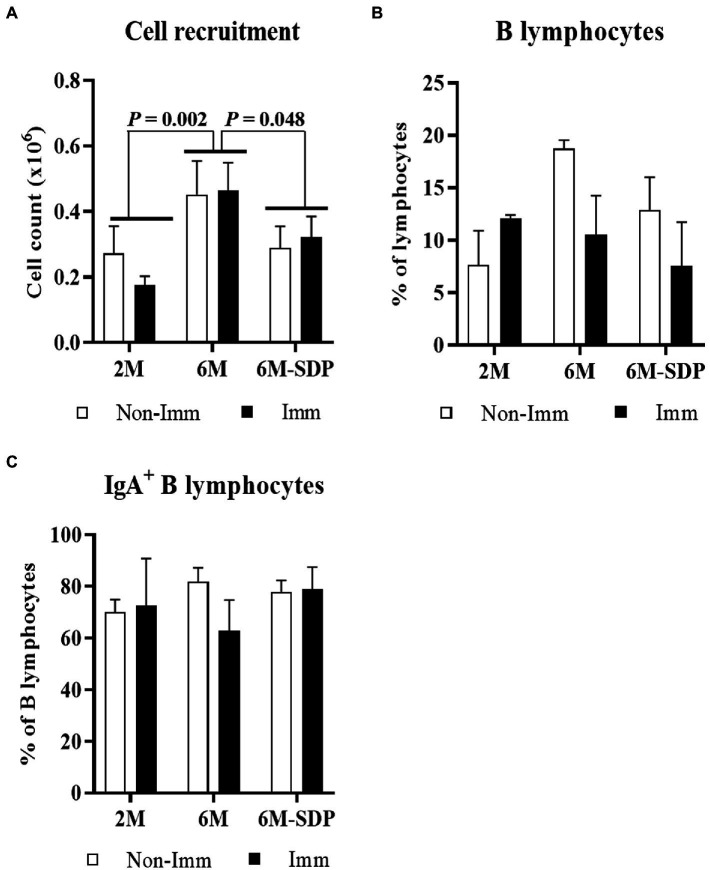
Cell count **(A)**, percentage of B lymphocytes **(B)**, and percentage of IgA^+^ B lymphocytes **(C)** in bronchoalveolar lavage fluid (BALF). Open bars represent non-immunized mice (**Non-Imm**); solid bars represent immunized mice (**Imm**). Results are expressed as means ± SEMs (*n* = 9–10 animals). Statistics: two-way ANOVA (age-feed and immunization factors; Fisher multiple comparison test). IgA, immunoglobulin A; SDP, spray-dried porcine plasma.

The leukocyte number in BALF was higher in aged mice than in young mice (6M vs. 2M; *p* = 0.002, Figure 4A). SDP supplementation prevented the effect of senescence on BALF cell counts (6M-SDP vs. 6M; *p* = 0.048) while immunization did not modify it. The percentages of B and IgA^+^ B lymphocytes in BALF were not modified by aging, feed, or immunization (Figures 4B,C, respectively).

#### 3.2.3. IgA concentration in the intestinal tract

**Figure 5:**
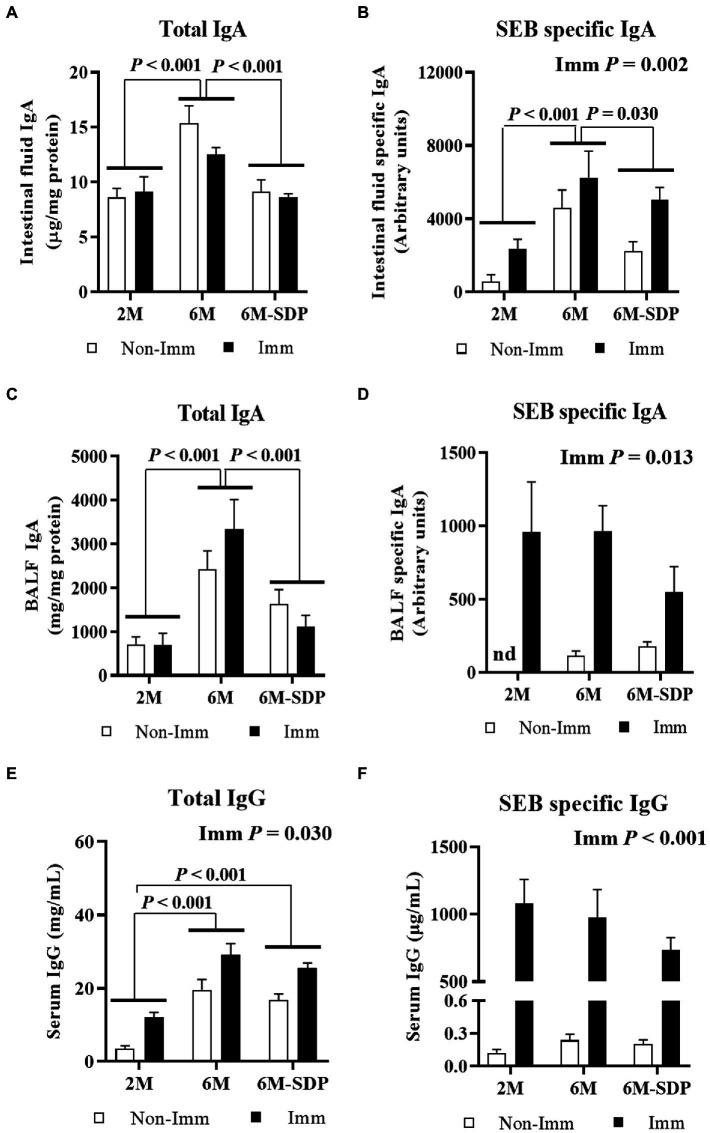
Immunoglobulins concentration. Total IgA concentration **(A)** and SEB-specific IgA titers **(B)** in intestinal fluid. Total IgA concentration **(C)** and SEB-specific IgA titers **(D)** in bronchoalveolar lavage fluid. Total IgG **(E)** and SEB-specific IgG **(F)** concentrations in serum. Open bars represent non-immunized (**Non-Imm**) mice; solid bars represent immunized (**Imm**) mice. Results are expressed as means ± SEMs (*n* = 9–10 animals). Statistics: two-way ANOVA (age-feed and immunization factors; Fisher multiple comparison test). BALF, bronchoalveolar lavage fluid; IgA, immunoglobulin A; SDP, spray-dried porcine plasma.

Aged mice had a higher concentration of total IgA in the intestinal lumen compared with young mice (*p* < 0.001, Figure 5A), and SDP supplementation prevented this effect. Immunization did not change the concentration of total IgA in intestinal fluid. Senescence augmented the amount of SEB-specific IgA in the intestinal lumen (*p* < 0.001, Figure 5B), which was diminished by SDP supplementation (*p* = 0.012). Immunization with SEB increased the concentration of SEB-specific IgA in the luminal content (*p* = 0.002).

#### 3.2.4. IgA concentration in BALF

Senescence increased the concentration of total IgA in BALF (*p* < 0.001, Figure 5C), which was reduced in senescent mice administered SDP feed (*p* < 0.001). Immunization did not modify this variable but greatly increased the concentration of SEB-specific IgA in BALF (*p* = 0.013, Figure 5D). There was no effect of age or SDP supplementation on the concentration of SEB-specific IgA.

#### 3.2.5. IgG concentration in serum

Senescent mice had a higher concentration of IgG in serum (6M vs. 2M; *p* < 0.001, Figure 5E), independently of the experimental feed consumed. Immunization increased the total IgG concentration in all groups (*p* = 0.030). The titer of SEB-specific IgG in serum was similar among experimental groups and increased after immunization in all groups (*p* < 0.001, Figure 5F).

### 3.3. Experiment 2: Lethal shock induction

#### 3.3.1. Survival rate to lethal shock with SEB and LPS

**Figure 6:**
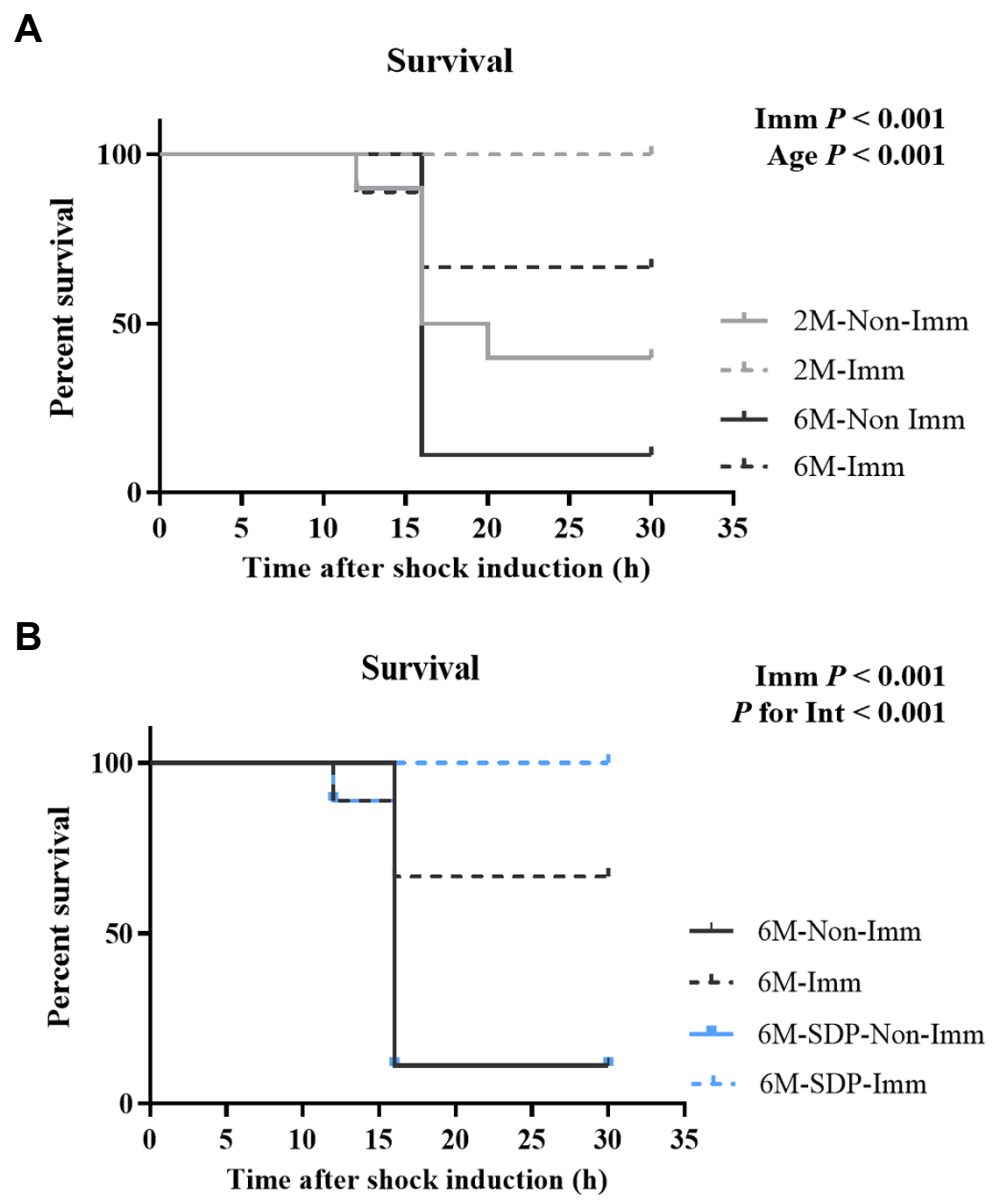
Survival rates of mice exposed to a lethal shock with SEB and LPS. Panel **(A)** compares 2-month-old (2 M) mice and 6-month-old mice fed control feed (6 M). Panel **(B)** compares 6-month-old mice fed control feed and 6-month-old mice fed SDP feed (6 M-SDP). Results are expressed as percent of survival (*n* = 9–10 animals). Statistics: log-rank (Mantel-Cox) test. Int indicates the interaction between immunization (Imm) and age-feed factors. LPS, lipopolysaccharide; SDP, spray-dried porcine plasma; SEB, *S. aureus* enterotoxin B.

All groups received a lethal shock *via* the administration of SEB and LPS to determine the degree of immune protection provided by SEB immunization. Young mice had a higher survival rate compared with senescent mice (*p* < 0.001, Figure 6A) and immunization completely protected young mice from the lethal shock (*p* < 0.001). Immunization also protected senescent mice from the shock (*p* < 0.001, Figure 6B), and it showed higher efficacy in senescent animals supplemented with SDP (*P* for interaction<0.001).

#### 3.3.2. Pro-inflammatory cytokines in intestinal mucosa and lung tissue after lethal shock

**Figure 7:**
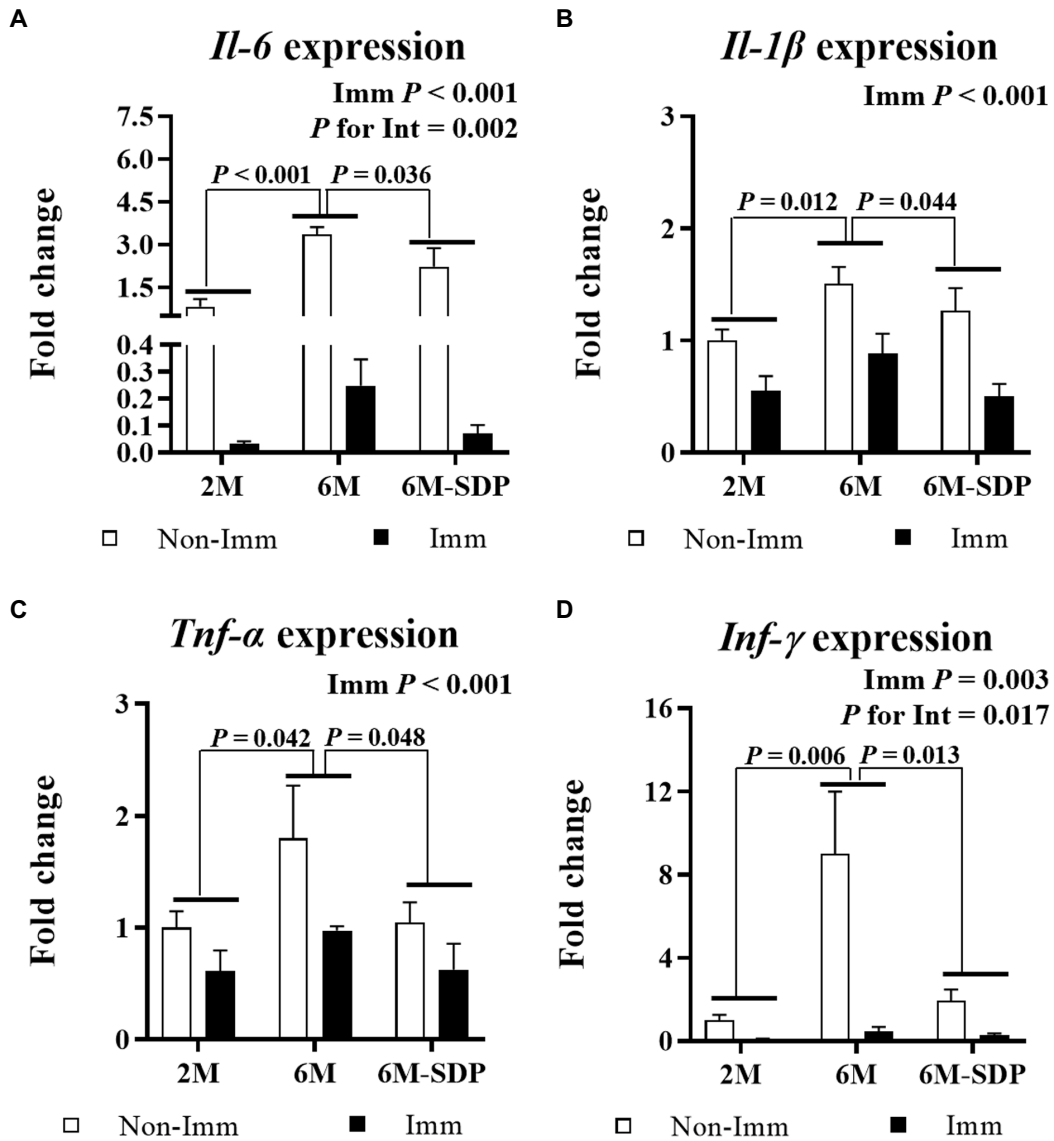
mRNA expression of *Il-6* **(A)**, *Il-1β* **(B)**, *Tnf-α* **(C)**, and *Ifn-γ* **(D)** in the jejunal mucosa. Open bars represent non-immunized (**Non-Imm**) mice; solid bars represent immunized (**Imm**) mice. All target genes were normalized to *Gusβ* expression. The fold-change expression is against immunized 2 M mice. Results are expressed as means ± SEMs (*n* = 9–10 animals). Statistics: two-way ANOVA (age-feed and immunization factors; Fisher multiple comparison test). Int indicates the interaction between immunization and age-feed factors. Gusβ, glucuronidase beta; Ifn-γ, interferon gamma; Il, interleukin; SDP, spray-dried porcine plasma; Tnf-α, tumor necrosis factor alpha.

Immunization protected mice from excessive pro-inflammatory cytokine production in the intestinal mucosa during lethal shock in all groups (all *p* < 0.05, Figure 7). Both immunized and non-immunized senescent mice exhibited increased expression of the pro-inflammatory cytokines *Il-6*, *Il-1β*, *Tnf-α*, and *Ifn-γ* in the jejunal mucosa compared with young mice (all *p* < 0.05). Senescent mice fed the SDP-supplemented feed had lower expression of all of them (all *p* < 0.05). Furthermore, immunization showed a greater protective effect on *Il-6* and *Ifn-γ* expression in animals supplemented with SDP (both *p* for interaction <0.05).

**Figure 8:**
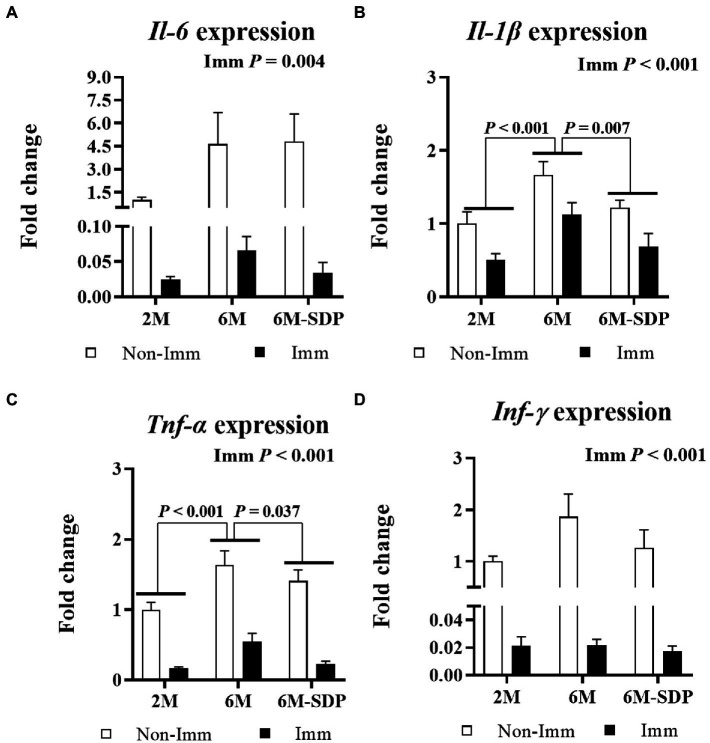
mRNA expression of *Il-6* **(A)**, *Il-1β* **(B)**, *Tnf-α* **(C)**, and *Ifn-γ* **(D)** in lung tissue. Open bars represent non-immunized (**Non-Imm**) mice; solid bars represent immunized (**Imm**) mice. All target genes were normalized to *Gusβ* expression. The fold-change expression is against immunized 2 M mice. Results are expressed as means ± SEMs (*n* = 9–10 animals). Statistics: two-way ANOVA (age-feed and immunization factors; Fisher multiple comparison test). Int indicates the interaction between immunization and age-feed factors. Gusβ, glucuronidase beta; Ifn-γ, interferon gamma; Il, interleukin; SDP, spray-dried porcine plasma; Tnf-α, tumor necrosis factor alpha.

With respect to lung tissue, immunization protected mice from the excessive production of pro-inflammatory cytokines in response to lethal shock in all groups (all *p* < 0.005, Figure 8). Both immunized and non-immunized senescent mice had increased expression of the pro-inflammatory cytokines *Il-1β* and *Tnf-α* in lung tissue compared with young mice (both *p* < 0.001). Senescent mice receiving the SDP-supplemented feed exhibited lower expression of them in response to lethal shock than animals receiving the control feed (both *p* < 0.05).

#### 3.3.3. Anti-inflammatory cytokines in intestinal mucosa and lung tissue after lethal shock

**Figure 9:**
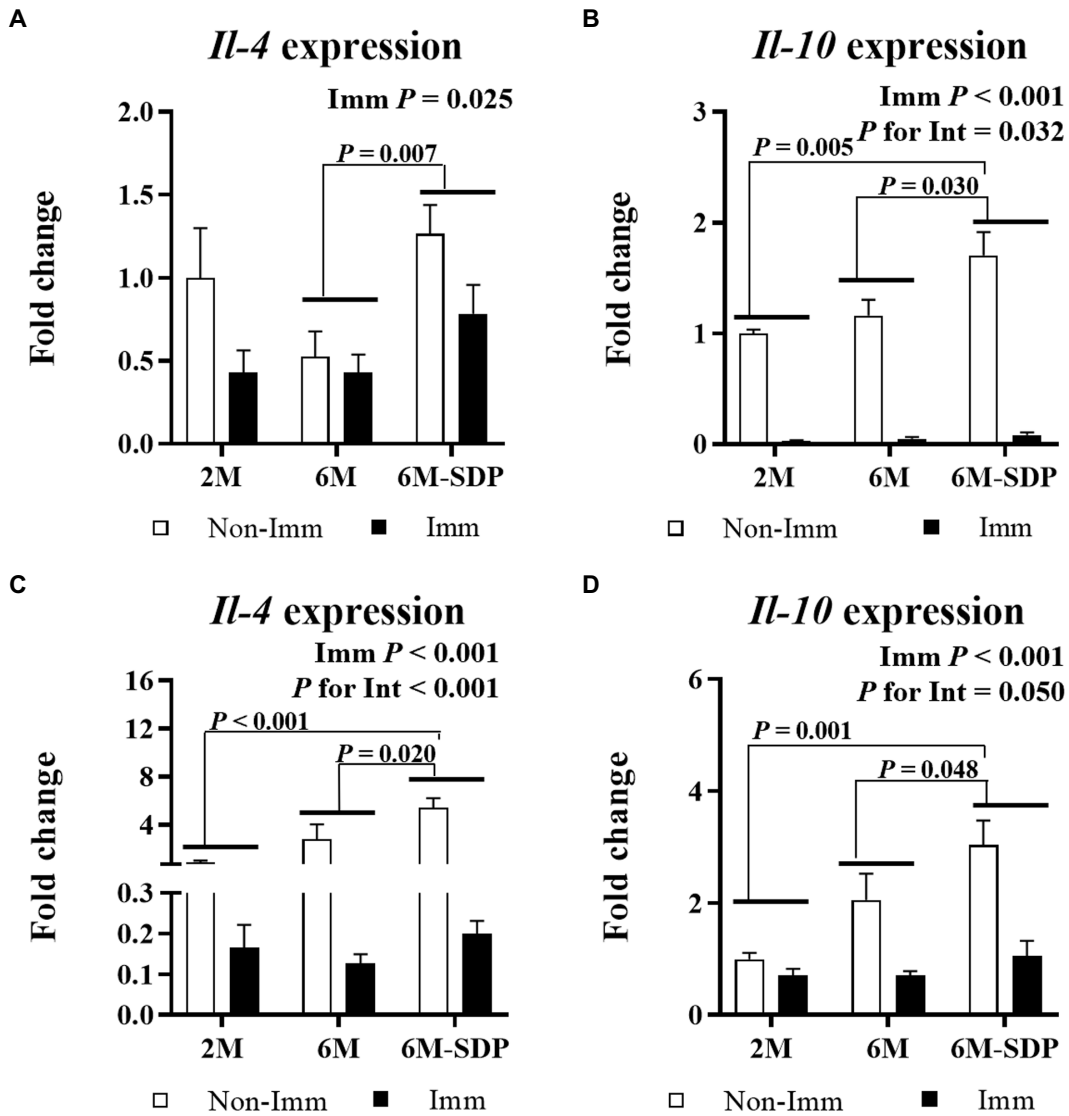
mRNA expression of *Il-4* and *Il-10* in the jejunal mucosa (**A** and **B**, respectively) and in lung tissue (**C** and **D**, respectively). Open bars represent non-immunized (**Non-Imm**) mice; solid bars represent immunized (**Imm**) mice. All target genes were normalized to *Gusβ* expression. The fold-change expression is against immunized 2 M mice. Results are expressed as means ± SEMs (*n* = 9–10 animals). Statistics: two-way ANOVA (age-feed and immunization factors; Fisher multiple comparison test). Int indicates the interaction between immunization and age-feed factors. Gusβ, glucuronidase beta; Il, interleukin; SDP, spray-dried porcine plasma.

Immunization reduced the expression of *Il-4* and *Il-10* in jejunum mucosa and lung tissue (both *p* < 0.05, Figure 9). Senescent mice supplemented with SDP showed higher expression of both cytokines in both tissues compared with mice fed control feed (all *p* < 0.05).

## 4. Discussion

Older people have a persistent low-intensity pro-inflammatory profile called inflammaging (1), which impairs pathogen-specific responses and the generation of effective vaccine protection (3). Some dietary supplements can modulate the gut barrier and the mucosal immune system, enhancing protection against infection (24) and vaccine response (25, 26). We therefore examined whether supplementation with SDP, which exhibits remarkable anti-inflammatory properties (10, 27), improved immune protection following mucosal immunization in aged mice. In this work, we demonstrate for the first time, that SDP supplementation can enhance vaccine protection in senescent mice.

Under our conditions, senescent mice showed a reduction in both the absolute number of cells in Peyer’s patches and the proportion of B lymphocytes, which were unchanged after immunization. These results are consistent with the widely described mucosal immunosenescence that occurs during aging (2, 28). Moreover, this reduction in the number and percentage of B lymphocytes at the intestinal level did not translate into lower IgA secretion, in line with the findings of McDonald et al. (29), where immunized senescent mice exhibited increased IgA levels, despite the reduction in B lymphocytes. Surprisingly, non-immunized senescent mice presented higher intestinal secretion of anti-SEB-specific IgA, which would indicate exposure to this microorganism prior to immunization. This could be due to an increased presence of *S. aureus* in the intestinal microbiota, because dysbiosis associated with aging leads to higher levels of pathogenic and opportunistic microorganisms (30), including *S. aureus* (31). SDP supplementation did not modify the percentage of B lymphocytes in Peyer’s patches, which is consistent with previous results in young rats (32). In fact, in that study, SDP did not modify the percentage of this population either in non-inflamed rats or in those challenged with SEB. In parallel, in the same experimental model, SDP supplementation reduced the percentage of activated Th lymphocytes and the production of proinflammatory cytokines (27), resulting in a reduced inflammatory response. Moreover, animals supplemented with SDP are less exposed to pathogenic or opportunistic microorganisms due to its prebiotic effects (33), which may contribute to the lower production of SEB-specific IgA, as observed in the animals supplemented with SDP.

In our study, senescence increased IgA secretion in the lungs of control mice, although the percentage of B lymphocytes did not change. This is consistent with the findings of McDonald et al. (29), where senescent mice exhibited increased IgA production but lower levels of B lymphocytes. On the other hand, aged mice showed increased immune cell recruitment into bronchoalveolar fluid, probably due to inflammation resulting from inflammaging (34). Dietary supplementation with SDP prevented leukocyte recruitment into the alveolar space of senescent mice, likely due to its anti-inflammatory activity and similar to what was previously observed in young animals with LPS-induced lung inflammation (21, 35).

At the systemic level, vaccination induces an IgG antibody-mediated immune response to control a potential infection. Indeed, increased levels of IgG are associated with effective immunization (36). Thus, the immunization increased the blood concentration of total and specific anti-SEB Igs in both young and senescent mice. These results are consistent with those obtained by Stiles et al. (19), in which they showed that nasal immunization with SEB increased the presence of specific anti-SEB Igs in the saliva. These data demonstrate that the mucosal immunization performed in this study not only protects mucosal areas, but also contributes to systemic protection.

To test the protective ability of immunization, animals were exposed to an LPS-enhanced SEB challenge involving the intraperitoneal administration of both toxins, because this is a widely accepted lethal model of SEB (37). Potentiating agents such as LPS are used to amplify the toxic effects of SEB because SEB has a lower affinity for MHC II molecules in mice compared with humans (38). Immunization increased the production of specific anti-SEB Igs under all conditions, helping to protect the mice against the lethal shock, and the immunized mice showed a higher survival rate than the non-immunized animals. These results are consistent with those of another model of SEB vaccination in which immunization also improved the survival rate of mice (39). Furthermore, immunized senescent mice fed the SDP feed had a higher survival rate than those fed the control feed. Therefore, SDP supplementation enhanced the immunization in SAMP8 mice.

Challenge with LPS-enhanced SEB in non-immunized mice markedly increased the expression of various pro-inflammatory cytokines, such as *Il-6*, *Tnf-α*, *Ifn-γ*, and *Il-1β*, in intestinal mucosa and only *Tnf-α*, and *Il-6* in lung tissue. This elevated cytokine production is a characteristic of the lethal shock and prolongs the inflammatory process, leading to acute mortality (40). Immunization reduced the pro-inflammatory mucosal cytokine storm, significantly improving the survival rate. Lethal challenge with SEB induced a more intense inflammatory response in aged mice than in young animals, probably due to the pro-inflammatory state that accompanies aging (1) and which we had also previously observed in this mouse model (11). On the other hand, senescent mice supplemented with SDP showed a lower intensity of the pro-inflammatory response in intestinal and lung tissue, improving the effectiveness of the immunization protection. Indeed, supplementation with SDP reduces the basal inflammation associated with senescence, allowing a more effective immune response (11). This reduction in the inflammatory response is vital to prevent the toxic effects of the toxin because pro-inflammatory mediators play essential roles in the lethal shock (40). Likewise, we observed a similar effect in this work, where senescent animals supplemented with SDP had a better protective immune response, showing a lower intensity of the inflammatory response to the lethal shock and an increased survival rate with respect to senescent animals fed the control feed. In addition, many strategies to protect mice against SEB have focused on inhibiting the inflammatory response. Examples of such strategies include attempts to block the activation of T cells (41), which are the main producers of these cytokines, to prevent the interaction of SEB with MHC II (42), to neutralize pro-inflammatory cytokines with antibodies (41), and to administer IL-10 to inhibit cytokine production by Th1 cells (43).

Furthermore, supplementation with SDP promoted the increase in *Il-4* expression in both the intestinal mucosa and lung tissue. This fact is remarkable because, in addition to being an anti-inflammatory cytokine and hallmark of Th2 differentiation (44), IL-4 has recently been described to be able to reduce the pathogenic potential of Th1 cells (45). Indeed, polarized Th2 cells, instead of inhibiting the Th1 phenotype, help to dampen the Th1 response by inducing the anti-inflammatory cytokine IL-10 (45). This cytokine limits the immune response to self and foreign antigens, protecting the host from excessive Th1 activation and preventing tissue damage (46). In addition, IL-10 expression in Th1 cells increases in response to a high dose of antigen (47).

Spray-dried plasma is a complex mixture of many functional components such as albumin, immunoglobulins, transferrin, fibrinogen and many other active proteins, lipids, growth factors, biologically active peptides, enzymes, hormones, amino acids, and other factors that have biological activity systemically and/or within the intestine independent of their nutritional value (48). It is unclear which component of the SDP elicits immunomodulatory and anti-inflammatory effect that improve animal welfare; but it is likely that the diverse mixture of bioactive proteins or peptides contributes to these beneficial effects. The mechanism of action may involve multiple functional components acting synergistically. Furthermore, the effect of SDP may not be direct, as evidenced by its ability to modulate the microbiota profile, promoting genera and species with anti-inflammatory effects (33).

The protective effect of SDP is not limited to bacterial antigens such as SEB. Studies have shown that SDP can also enhance immune protection against viral antigens, such as African swine fever virus (ASFV) in young pigs (49). In addition, SDP supplementation has been found to promote an earlier antibody response to porcine epidemic diarrhea virus (PEDV) in pigs infected with PEDV, leading to reduced clinical disease, as well as decreased amount and duration of viral shedding during acute infection (50). While bacterial and viral infections elicit different immune responses, SDP supplementation seems to elicit a common response to vaccination, potentially modulating the mucosal immune response. Moreover, commensal bacteria are known to play a role in shaping the immune system tone, which may also influence vaccine efficacy (51). Recently, a study observed that potentiation of the innate immune response through Toll Like Receptor (TLR)-4 could enhance vaccine protection against influenza viruses in senescent mice (52). Given that SDP supplementation influence the intestinal microbiota, increasing TLR4 expression (33), this could be a mechanism underlying the improved vaccine protection observed in senescent mice supplemented with SDP.

In conclusion, in this study, we have established a model of nasal immunization in senescent mice that protected them mucosally and systemically, as reflected by increased levels of anti-SEB-specific Igs and a higher survival rate to lethal shock with SEB. Furthermore, dietary supplementation with SDP reduced the basal inflammation associated with aging, improving the efficacy of immunization in terms of the response to the lethal shock. The results of this study, combined with previous research, suggest that SDP can enhance the protection of vaccination by reducing the pro-inflammatory state associated with senescence. This finding opens up the possibility of using SDP to improve immune protection in the elderly population. However, it should be noted that this study was conducted only in males, and further research is needed to understand the level of protection provided by SDP in females, as well as the underlying mechanisms before it can be applied to humans. Nonetheless, given that SDP is already being used in farm animals, its potential use in domestic animals appears to be more feasible.

## Data availability statement

The raw data supporting the conclusions of this article will be made available by the corresponding author, without undue reservation.

## Ethics statement

The animal study was reviewed and approved by Ethics Committee for Animal Experimentation of the Universitat de Barcelona and the Catalan government (ref. 484/16 and 9272, respectively).

## Author contributions

LM, CA, JP, MM, and AP-B conceived and design the experiments. LM, CR-C, and AP-B conducted research and data analysis. JP contributed with essential reagents and materials. LM, CR-C, and AP-B wrote the original draft preparation. CA, JP, and MM review and edited the manuscript. All authors contributed to the article and approved the submitted version.

## Funding

This research was funded by APC-Europe S.L.U. (Granollers, Spain) by research contracts with the Bosch i Gimpera Foundation of the University of Barcelona. The research groups were also supported by grants 2017SGR945 for Consolidated Research Groups, Generalitat de Catalunya, Spain. CR-C was supported by a grant from the Bosch i Gimpera Foundation (Universitat de Barcelona). The authors are members of the Institut de Recerca en Nutrició, i Seguretat Alimentària (INSA-UB), which is recognized as a Maria de Maeztu Unit of Excellence and funded by MICIN/AEI/FEDER (CEX2021-001234-M).

## Conflict of interest

JP is employed by APC Europe S.L.U. (Granollers, Spain).

The remaining authors declare that the research was conducted in the absence of any commercial or financial relationships that could be construed as a potential conflict of interest.

## Publisher’s note

All claims expressed in this article are solely those of the authors and do not necessarily represent those of their affiliated organizations, or those of the publisher, the editors and the reviewers. Any product that may be evaluated in this article, or claim that may be made by its manufacturer, is not guaranteed or endorsed by the publisher.
